# Design and Biological Evaluation of Colchicine-CD44-Targeted Peptide Conjugate in an In Vitro Model of Crystal Induced Inflammation

**DOI:** 10.3390/molecules25010046

**Published:** 2019-12-21

**Authors:** Khalid A. Zoghebi, Emira Bousoik, Keykavous Parang, Khaled A. Elsaid

**Affiliations:** 1 Center for Targeted Drug Delivery, Department of Biomedical and Pharmaceutical Sciences, Chapman University School of Pharmacy, Harry and Diane Rinker Health Science Campus, Irvine, CA 92618, USA; zoghe101@mail.chapman.edu (K.A.Z.); bouso100@mail.chapman.edu (E.B.); 2 Department of Pharmaceutical Chemistry, College of Pharmacy, Jazan University, Jazan 82826, Saudi Arabia

**Keywords:** CD44, colchicine, gout, hyaluronic acid, MSU, colchicine-peptide conjugate

## Abstract

Gout is an inflammatory arthritis due to the joint deposition of monosodium urate (MSU) crystals. Phagocytosis of MSU crystals by tissue macrophages results in the generation of reactive oxygen species (ROS) and production of inflammatory cytokines and chemokines. Colchicine use in gout is limited by severe toxicity. CD44 is a transmembrane glycoprotein that is highly expressed in tissue macrophages and may be involved in gout pathogenesis. The P6 peptide is a 20-amino acid residue peptide that binds to CD44. We hypothesized that the conjugation of colchicine to the P6 peptide would reduce its off-target cytotoxicity while preserving its anti-inflammatory effect. A modified version of P6 peptide and colchicine-P6 peptide conjugate were synthesized using Fmoc/tBu solid-phase and solution-phase chemistry, respectively. A glutaryl amide was used as a linker. The P6 peptide was evaluated for its binding to CD44, association, and internalization by macrophages. Cytotoxic effects of P6 peptide, colchicine, and colchicine-P6 peptide on macrophages were compared and the inhibition of ROS generation and interleukin-8 (IL-8) secretion in MSU-stimulated macrophages treated with P6 peptide, colchicine, or colchicine-P6 peptide was studied. We confirmed that the P6 peptide binds to CD44 and its association and internalization by macrophages were CD44-dependent. Colchicine (1, 10, and 25 μM) demonstrated a significant cytotoxic effect on macrophages while the P6 peptide and colchicine-P6 peptide conjugate (1, 10 and 25 μM) did not alter the viability of the macrophages. The P6 peptide (10 and 25 μM) reduced ROS generation and IL-8 secretion mediated by a reduction in MSU phagocytosis by macrophages. The colchicine-P6 peptide significantly reduced ROS generation and IL-8 secretion compared to the P6 peptide alone at 1 and 10 μM concentrations. Conjugation of colchicine to the P6 peptide reduced the cytotoxic effect of colchicine while preserving its anti-inflammatory activity.

## 1. Introduction

Gout is a prevalent inflammatory arthritis due to the precipitation of serum uric acid into crystallized deposits of monosodium urate (MSU) crystals in and around the joint [1,2,3]. On a worldwide level, it is estimated that between 1 and 3% of males and females, respectively, between the ages of 40 and 60 years old suffer from gout [4]. Gout flares are characterized by acute episodes of intense pain, severe inflammation, and joint swelling, with the knee and metatarsophalangeal joints most frequently affected [1,3]. Management of acute gout flares remains suboptimal, and hospitalizations due to poorly controlled flares have significantly increased over the past two decades [5,6,7,8]. Treatment modalities of acute gout flares include the use of nonsteroidal anti-inflammatory drugs (NSAIDs), selective cycloxygenase-2 (COX2) enzyme inhibitors, colchicine, and corticosteroids [9,10]. However, the use of these agents is not without harm, given the comorbidities seen in older patients with gout [11,12,13].

The normal synovium contains two types of intimal cells: type A macrophages and type B synovial fibroblasts [14]. During an acute episode of gouty arthritis, deposition of MSU crystals in joints stimulates resident macrophages to produce the inflammatory cytokine interleukin-1 beta (IL-1β) and inflammatory chemokines, interleukin-8 (IL-8), and monocyte chemoattractant protein-1 (MCP-1) [15,16]. The secretion of IL-8 results in the recruitment of inflammatory cells (e.g., neutrophils and monocytes) to the affected joints [16,17,18]. This inflammatory cascade is mediated by phagocytosis of MSU crystals by joint resident macrophages [19,20]. The mechanism of phagocytosis is not entirely understood and the toll-like pattern recognition family of receptors (TLRs) may play a role in facilitating crystal phagocytosis [21,22]. Subsequent to MSU crystal phagocytosis, reactive oxygen species (ROS) are generated through the activation of NADPH oxidase and downstream activation of NLRP3 inflammasome and nuclear factor kappa-b (NF-κB), resulting in the expression and production of IL-1β and IL-8 [23,24,25].

The cluster determinant-44 (CD44) receptor is a single pass transmembrane glycoprotein that has various functions in cell division, migration, adhesion, and signaling [26]. CD44 expression level in the synovium may be linked to the inflammatory status of the joint with increased expression of certain isoforms over the course of experimental arthritis and in patients with advanced osteoarthritis (OA) [27,28,29]. The CD44 receptor may also fulfill a phagocytic function in immune cells [30]. A number of endogenous ligands to CD44 have been identified including high molecular weight hyaluronic acid (HA) and the glycoprotein, proteoglycan-4 (PRG4), two major components of joint synovial fluid (SF) [31]. Binding of HA and PRG4 to CD44 on the surface of macrophages was shown to result in ligand internalization [32,33]. Furthermore, we have previously shown that recombinant human PRG4 inhibits MSU phagocytosis by macrophages in a mechanism that may involve CD44 and/or TLR receptor interactions [33]. The utility of CD44 as a targeting receptor on macrophages was also reported by utilizing HA-coated nanoparticles to repolarize inflammatory macrophages to an anti-inflammatory phenotype [34,35].

Colchicine is a natural alkaloid that is used in the management of acute gout flares as well as prophylaxis against acute gout attacks within six months of initiating chronic urate-lowering therapy [36]. In addition to its use in gout, colchicine has also shown clinical efficacy in reducing pain and inflammation in symptomatic OA, treatment of inflammation in crystal-induced chondrosis due to deposition of calcium pyrophosphate crystals, and may also have a clinical benefit in lowering cardiovascular risk in patients with coronary artery disease [36,37]. Due to its ability to interfere with microtubule assembly, colchicine can control numerous cell functions, including cytokine and chemokine secretion and inflammatory cell migration and proliferation [36]. This microtubule disrupting effect was also shown to block cancer cells in the G2/M phase, thus its cytotoxic effect may prove useful for cancer treatment [38]. The therapeutic effect of colchicine on macrophages in the context of MSU induced inflammation was shown to lie upstream of microtubule disruption via inhibition of ROS generation [39]. Due to its off-target cytotoxicity, oral colchicine use is limited by its gastrointestinal toxicity and drug–drug interactions, particularly in patients with renal and hepatic impairments resulting in toxic outcomes (e.g., myopathy and liver damage) [36,37]. In addition, fatalities have been reported due to both accidental and therapeutic colchicine poisoning [40].

Localized drug delivery into the joint is one potential approach to enhance the joint residence time of injected therapeutics and reduce systemic exposure of administered therapeutics. The joint half-life estimates of small molecules and recombinant proteins following intra-articular administration is in the order of hours due to rapid systemic clearance [41,42]. Encapsulation of small molecules and proteins in microspheres offers the advantage of extending their joint residence time and reducing systemic exposure [43,44]. Given the complexity of formulating microsphere delivery systems, peptide-drug conjugates (PDC) may offer an outstanding alternative approach to deliver small molecules selectively to cells or specific targets, thereby enhancing their efficacy and reducing off-target interactions. PDCs represent an important class of prodrugs that are formed through covalent binding of a specific peptide sequence to a drug via a cleavable linker [45]. Given its importance in inflammatory joint disease, CD44 receptor targeting may offer a unique opportunity to deliver intra-articular therapeutics for the treatment of joint inflammatory diseases. The P6 peptide, FDAIAEIGNQLYLFKDGKYW, is a novel CD44-binding peptide that was identified from studying the interaction of pro matrix metalloproteinase-9 (pro-MMP-9) with CD44 as it relates to the proliferation of chronic lymphocytic leukemia (CLL) cells [46]. The P6 peptide was shown to effectively prevent pro-MMP-9 CD44-dependent binding to CLL cells [46].

The overall objective of this study was to evaluate the feasibility of conjugating the P6 peptide to *N*-deacetylcolchicine via a hydrolyzable linker and study the efficacy and safety of the colchicine-P6 peptide conjugate in an in vitro model of MSU stimulated macrophages. We hypothesized that conjugation of colchicine to the P6 peptide will modify colchicine’s intracellular distribution, thereby reducing its cytotoxic effect while preserving its anti-inflammatory efficacy. We assessed the anti-inflammatory efficacy using a combination of IL-8 secretion and ROS generation. We initially studied CD44 expression in macrophages and characterized the role of CD44 in mediating MSU crystal uptake by macrophages. We also evaluated the affinity of P6 binding toward the CD44 receptor using HA as a comparator. We studied the association and internalization of fluorescently-labeled P6 peptide in human THP-1 macrophages. Subsequently, we studied the impact of P6 peptide, colchicine, and colchicine-P6 peptide treatments on MSU phagocytosis by macrophages, macrophage cell viability, production of IL-8, and ROS generation. Finally, we evaluated the stability of the P6 peptide in SF aspirates from donors with no history of degenerative joint disease.

## 2. Results and Discussion

### 2.1. Synthesis of P6 Peptide

**Scheme 1:**
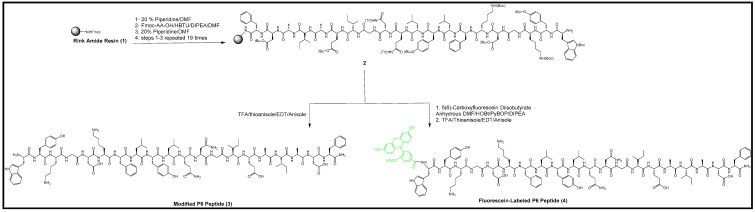
Synthesis of the resin-attached peptide (2), modified P6 peptide (3), and fluorescein-labeled P6 peptide (4) by solid-phase peptide synthesis.

Modified P6 peptide H_2_N-WYKGDKFLYLQNGIEAIADF-CO-NH_2_ in reverse order was assembled on a Fmoc Rink amide resin using Fmoc solid-phase synthesis starting with Fmoc-L-phenylalanine (Fmoc-Phe-OH). Fmoc deprotection was done by adding 20% piperidine in dimethylformamide (DMF) (*v*/*v*). The modified version of the P6 peptide may not exhibit a similar behavior, even if all of the amino acids and side chains in the sequence are the same. The subsequent Fmoc-protected amino acids were coupled to the peptide template until the last amino acid Fmoc-Trp(Boc)-OH was coupled. The final Fmoc deprotection was carried out to make a free N-terminal tryptophan residue for the conjugation of colchicine, as shown in Scheme 1. After finishing the synthesis of the 20-amino acid sequence of the P6 peptide, the linear peptide was confirmed by mass spectrometry analysis (Figure S1, Supplementary Materials).

### 2.2. Synthesis of Colchicine-Glutaryl Amide Conjugate

**Scheme 2:**
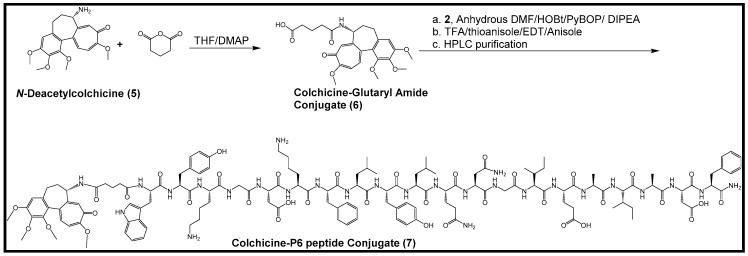
Synthesis of colchicine-glutaryl amide (6) and conjugation of the colchicine-glutaryl amide conjugate with the P6 peptide (7).

Coupling of *N*-deacetylcolchicine with glutaric anhydride was carried out through solution-phase chemistry in the presence of anhydrous tetrahydrofuran, 4-dimethylaminopyridine (DMAP) and triethylamine (TEA) as a base (Scheme 2). The compound was used in conjugation with the resin-attached P6 peptide to afford the desired conjugate with the P6 peptide. The formation of the desired compound was confirmed by Quadrupole Time-of-flight Mass Spectrometry (Q-TOF) (Figure S2, Supplementary Materials).

### 2.3. Synthesis of Colchicine-P6 Peptide Conjugate

The peptide-attached resin was used in the coupling with a colchicine-glutaryl amide conjugate synthesized by the solution phase chemistry in the presence of benzotriazol-1-yl-oxytripyrrolidinophosphonium hexafluorophosphate (PyBOP), hydroxybenzotriazole (HOBt), and *N*,*N*-diisopropylethylamine (DIPEA). The cleavage cocktail containing trifluoroacetic acid (TFA), thioanisole, 1,2-ethanedithiol (EDT), and anisole (90:5:3:2 *v*/*v*/*v*/*v*) was used to cleave the resin and all side-chain protecting groups from the conjugate. After purification via reversed-phase high performance liquid chromatography (HPLC) and lyophilization, the structure of the conjugate was confirmed by high-resolution matrix-assisted laser desorption/ionization time-of-flight (MALDI-TOF) analysis (Figure S3, Supplementary Materials).

### 2.4. Synthesis of Fluorescein-Labeled P6 Peptide

Scheme 1 also depicts the chemical reaction between the P6 peptide-attached resin and 5(6)-carboxyfluorescein diisobutyrate. Protected P6 peptide-attached resin was used in the coupling with 5(6)-carboxyfluorescein diisobutyrate on a solid support in the presence of PyBOP, HOBt, and DIPEA. After the purification of the crude peptide via reversed-phase HPLC and lyophilization, the structure of the biotinylated P6 peptide was confirmed by high-resolution MALDI-TOF analysis (Figure S4, Supplementary Materials).

### 2.5. Characterization of CD44 Receptor Expression on Macrophages, Regulation under Conditions of Inflammation and Role of CD44 in Modulating Monosodium Urate (MSU) Crystal Phagocytosis by Macrophages

**Figure 1:**
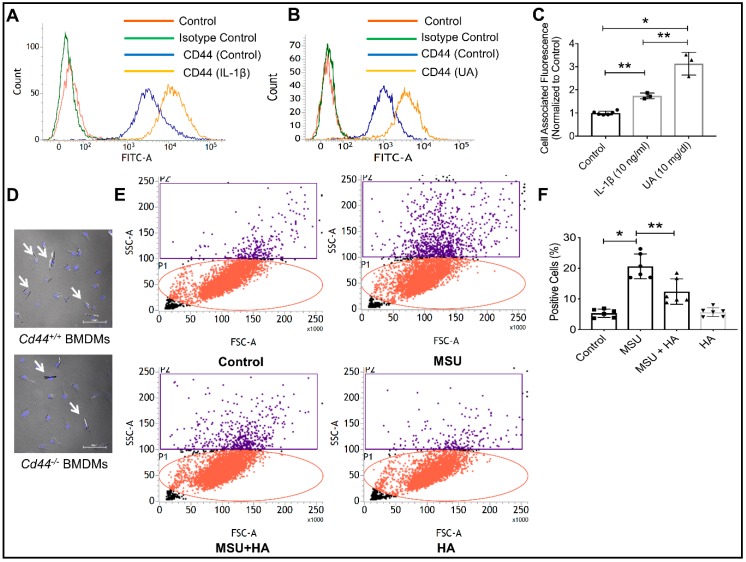
The CD44 receptor is highly expressed on differentiated human THP-1 macrophages, is induced by proinflammatory cytokine interleukin-1 beta (IL-1β) and soluble uric acid (UA) and is directly involved in the phagocytosis of monosodium urate (MSU) crystals. * p < 0.001; ** p < 0.01. Data are presented as scatter plots of 3–5 independent experiments with mean and standard deviations highlighted. (**A**) A representative flow cytometry plot showing the expression of CD44 receptor on THP-1 macrophages and the impact of IL-1β (10 ng/mL) treatment on CD44 receptor protein expression. A rightward shift was observed indicative of increased receptor density on cell surface. (**B**) A representative flow cytometry plot showing enhanced CD44 receptor localization on THP-1 macrophages following treatment with UA (10 mg/mL). (**C**) CD44 receptor staining was significantly higher in IL-1β and UA-treated macrophages compared to untreated cells. (**D**) MSU crystals were detected intracellularly to a greater extent in bone marrow-derived macrophages (BMDMs) from CD44 competent animals (*Cd44^+/+^*) compared to BMDMs from CD44 knockout animals (*Cd44^−/−^*). (**E**) Representative flow cytometry plots depicting increased cell side-scatter of THP-1 macrophages as a result of MSU phagocytosis. Treatment with the CD44 receptor-ligand hyaluronic acid (HA; 100 μg/mL) reduced MSU phagocytosis indicated by a reduction in macrophage cell side scatter. (**F**) HA treatment significantly reduced MSU phagocytosis by THP-1 macrophages.

Human THP-1 macrophages express the CD44 receptor as shown by flow cytometry (Figure 1A). CD44 surface expression increased when THP-1 macrophages were treated with IL-1β (10 ng/mL) (*p <* 0.01) or soluble uric acid (UA; 10 mg/dL) (*p <* 0.001) compared to the untreated control macrophages (Figure 1A,B). The magnitude of enhancement in the cell surface CD44 protein staining subsequent to IL-1β was approximately two-fold compared to approximately 3-fold enhancement with UA treatment. UA treatment increased CD44 protein expression by THP-1 macrophages compared to IL-1β treatment (*p <* 0.01, Figure 1C). This is a significant finding as patients with chronic gout often have persistent hyperuricemia and detectable IL-1β levels in inflamed joints [47]. Given that the CD44 receptor is upregulated under conditions of inflammation specific to gout, CD44 is plausibly an attractive candidate for targeting intracellular therapeutics for intra-articular administration that would otherwise be rapidly cleared from the joint [41]. We have further investigated the direct role that CD44 may play in facilitating MSU crystal uptake by macrophages. Bone marrow-derived macrophages (BMDMs) from CD44 wild-type animals (*Cd44^+/+^*) appeared to internalize MSU crystals to a greater extent when compared to the CD44 knockout (*Cd44^−/−^*) animals (white arrows point to MSU crystals in DAPI-stained BMDMs; Figure 1D). This observation is consistent with the phagocytic role of the CD44 receptor and validates our earlier observations that PRG4, a CD44 ligand, prevents MSU phagocytosis by macrophages [33]. Using flow cytometry to detect MSU phagocytosis via a change in macrophage cell side-scatter [33], we observed that the CD44 receptor-ligand, HA, significantly reduced MSU phagocytosis by THP-1 macrophages. Figure 1E depicts the representative flow cytometry plots of MSU-treated macrophages in the absence or presence of HA (100 μg/mL). Two regions of interest were identified: the P1 region (red), which represents the majority of macrophage cell population, and the P2 region (purple) with increased side-scattering as a result of MSU phagocytosis. MSU treatment appeared to increase the number of cells in the P2 region while HA co-treatment reduced the number of cells in the P2 region. We previously validated this indirect phagocytosis assay against a direct method of assessing crystal uptake by cells using cell counting under the microscope [33]. The quantitative analysis of the percentage of macrophage cell population in the P2 region across different experimental groups is shown in Figure 1F. HA significantly reduced MSU phagocytosis by THP-1 macrophages (*p <* 0.01; Figure 1F). The efficacy of HA in reducing MSU phagocytosis by macrophages is likely to be due to its ability to bind the CD44 receptor, resulting in receptor internalization [32]. This interaction reduced the number of available CD44 surface receptors and thus attenuated macrophage phagocytic activity against MSU crystals. Taken together, our findings establish the CD44 receptor as a promising therapeutic target in gout, both through indirectly interfering with MSU phagocytosis by macrophages and in facilitating the uptake of small molecules that have an intracellular target in macrophages.

### 2.6. Comparative Binding of P6 Peptide and HA to CD44 Receptor and CD44 Dependent Association of P6 Peptide with THP-1 Macrophages

**Figure 2:**
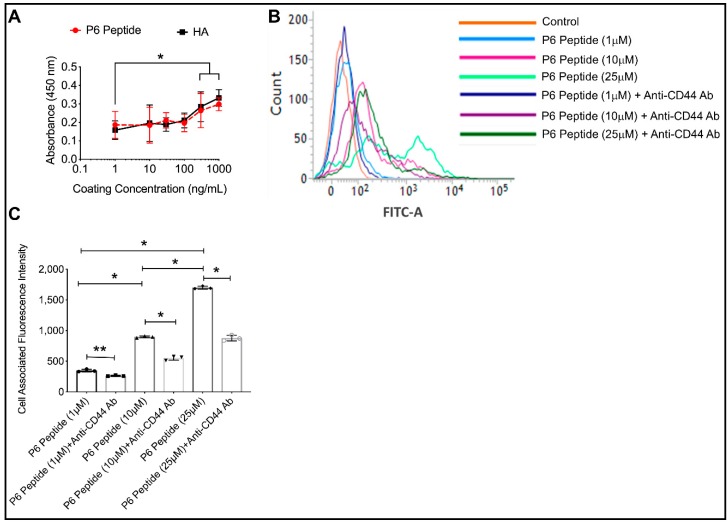
Comparative binding of the P6 peptide and high molecular weight hyaluronic acid (HA) to the recombinant human CD44 receptor using an enzyme-linked immunosorbent assay (ELISA) format and CD44-dependent association of fluorescein-P6 peptide with human THP-1 macrophages. Fluorescein-P6 peptide was incubated with THP-1 macrophages for 60 min in the absence or presence of an anti-CD44 antibody (Ab; 2.5μg/mL) and cell-associated fluorescence was determined by flow cytometry. Data are presented as the mean and standard deviation of three independent experiments. * p < 0.001; ** p < 0.01. (**A**) The P6 peptide and HA exhibited a concentration-dependent binding to the CD44 receptor with no difference in binding signals between the P6 peptide and HA across all coating concentrations. (**B**) A representative flow cytometry plot showing reduced association of the P6 peptide with THP-1 macrophages in the presence of an anti-CD44 Ab. (**C**) Neutralization of the CD44 receptor significantly reduced the P6 peptide association with THP-1 macrophages.

We studied the interaction between the P6 peptide and the CD44 receptor using a combination of an ELISA binding assay and the association of fluorescein-labeled P6 peptide with THP-1 macrophages. We compared the binding affinity of the P6 peptide to the CD44 receptor using HA as a positive control. Using a concentration range between 1 ng/mL and 1 μg/mL, the P6 peptide and HA demonstrated a concentration-dependent binding to the CD44 receptor (500 ng/mL and 1 μg/mL concentrations showed a higher binding compared to 1 ng/mL; *p <* 0.001; Figure 2A). Furthermore, there was no significant difference between the P6 peptide and HA’s binding to CD44. Using flow cytometry, we observed that fluorescein-P6 peptide associates with THP-1 macrophages over 1 h in a concentration-dependent manner (Figure 2B). In addition, pre-incubation of THP-1 macrophages with an anti-CD44 antibody reduced the association of fluorescein-P6 peptide with THP-1 macrophages (Figure 2B). The reduction in cell-associated fluorescence with the fluorescein-P6 peptide as a result of pre-incubation with anti-CD44 antibody was approximately 50% across the 1, 10, and 25 μM concentrations (Figure 2C). This finding indicates that the association of the P6 peptide with THP-1 macrophages was dependent on its binding to the CD44 receptor. The lack of the complete inhibition of P6 peptide association with the macrophages as a result of CD44 antibody-mediated receptor neutralization may be explained by the competition between the peptide and the antibody on binding to the receptor and/or the presence of unblocked CD44 receptors, even with antibody treatment. Nonetheless, other mechanisms of association cannot be ruled out. One potential surface receptor is the α4β1 integrin receptor to which pro-MMP-9 can bind [46].

### 2.7. Concentration-Dependent Internalization of Fluorescein-P6 Peptide in THP-1 Macrophages Using Confocal Microscopy

**Figure 3:**
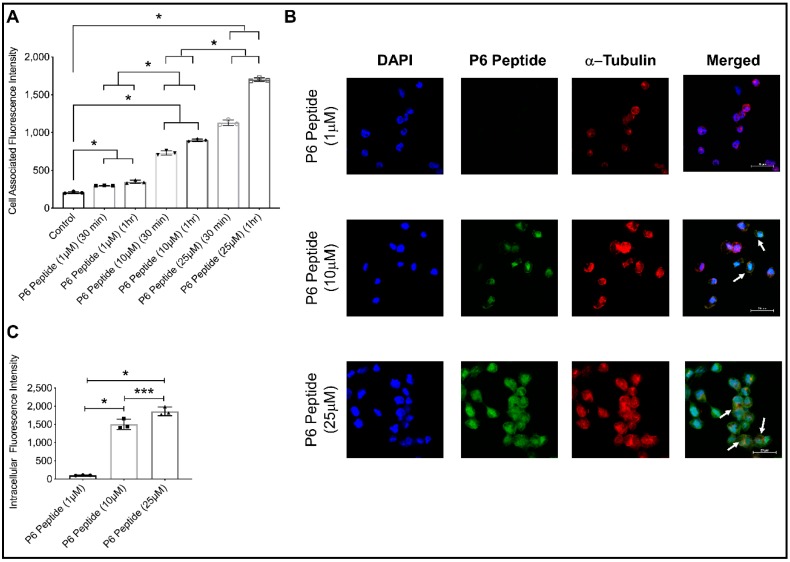
Time and concentration-dependent association of the fluorescein-P6 peptide with THP-1 macrophages and internalization of P6 peptide in macrophages following incubation for 6 h. THP-1 macrophages were counterstained for α-tubulin (red) and nuclei (blue). Data are presented as scatter plots of three independent experiments with the mean and standard deviation highlighted. * p < 0.001; *** p < 0.05. (**A**) Fluorescein-P6 peptide demonstrated a higher association with THP-1 macrophages at 1 h compared to 30 min incubation and higher association was observed at 25 μM compared to the 1 and 10 μM concentrations. (**B**) Representative confocal microscopy images showing fluorescein-P6 peptide (green), α-tubulin (red), and nuclear (blue) staining in THP-1 macrophages at 6 h. White arrows point to co-localization of the fluorescein-P6 peptide with α-tubulin, indicating internalization of the P6 peptide by macrophages. (**C**) The internalization of the P6 peptide in THP-1 macrophages was higher at the 25 μM concentration compared to the 1 and 10 μM concentrations.

The P6 peptide rapidly associated with the THP-1 macrophages in a concentration-dependent manner. This is evidenced by the higher mean cell-associated fluorescence observed with a 25 μM P6 peptide concentration compared to 1 and 10 μM concentrations (*p <* 0.001 for both comparisons; Figure 3A). We detected internalization of the P6 peptide in the THP-1 macrophages at the 10 and 25 μM concentrations (Figure 3B). The internalization was evident by co-localization of the P6 peptide with the cytoskeleton marker, α-tubulin. Using z-stacking, the mean intracellular green fluorescence in the THP-1 macrophages was determined. Mean intracellular fluorescence intensity was highest with the 25 μM concentration. In addition, mean intracellular fluorescence intensity of the 10 μM concentration was higher than the corresponding mean intensity of the 1 μM concentration (Figure 3C). Taken together, the P6 peptide is able to associate with THP-1 macrophages and is subsequently internalized by macrophages. The mechanism of association and uptake is CD44-dependent. Therefore, the P6 peptide can be successfully used to deliver conjugated small molecules into macrophages, especially under conditions that upregulate CD44 surface expression.

### 2.8. Cytotoxicity of P6 Peptide, Colchicine, and Colchicine-P6 Peptide Conjugate against Human THP-1 Macrophages

**Figure 4:**
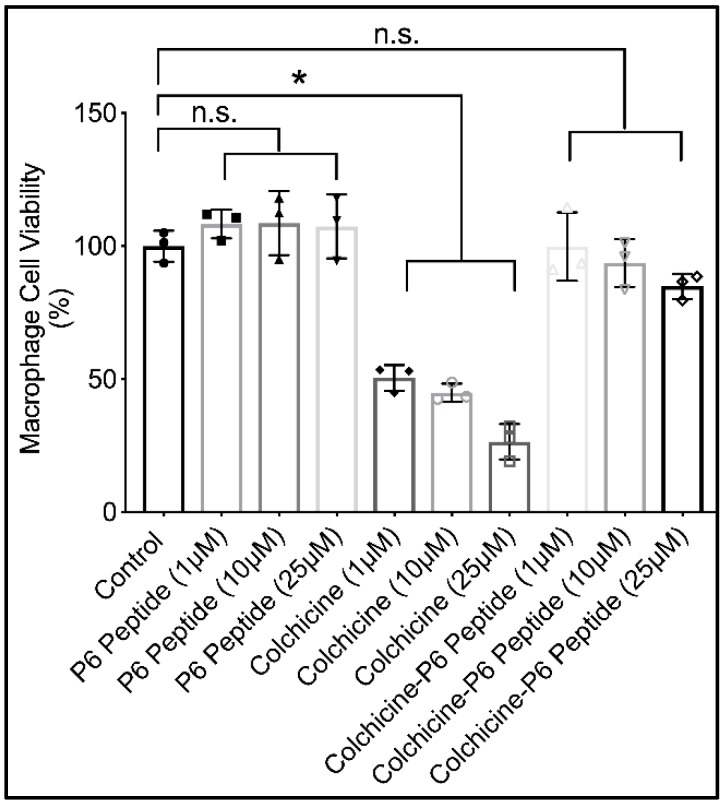
Impact of P6 peptide, colchicine, and colchicine-P6 peptide conjugate treatments on human THP-1 macrophage cell viability at 24 h using the Cell Counting Kit-8 (CCK-8) assay. P6 peptide and colchicine-P6 peptide treatments (1, 10, and 25 μM) showed no significant difference in cell viability when compared with the control. Colchicine (1, 10, and 25 μM) dose-dependently reduced macrophage cell viability. Data are presented as scatter plots of three independent experiments with at least duplicate wells per treatment. Mean and standard deviations are highlighted. * p < 0.001; n.s.: non-significant.

The impact of P6 peptide conjugation to colchicine on colchicine’s toxicity against THP-1 macrophages is shown in Figure 4. Colchicine demonstrated a dose-dependent cytotoxic effect against macrophages over 24 h. At the lowest concentration (1 μM), macrophage cell viability was reduced by approximately 50%. At the highest concentration (25 μM), macrophage cell viability was reduced by approximately 80%. In contrast, the P6 peptide demonstrated no cytotoxic effect against THP-1 macrophages at the same concentration range as those of colchicine. At 25 μM, colchicine-P6 peptide treatment resulted in a numerical reduction in macrophage cell viability by approximately 15%, compared with a reduction in cell viability of approximately 6% in 10 μM concentration. This numerical reduction did not reach statistical significance (*p =* 0.99 for control vs. colchicine-P6 peptide (10 μM); *p =* 0.48 for control vs. colchicine-P6 peptide (25 μM)). In addition, the percentage of viable cells in colchicine-P6 peptide-treated macrophages was significantly higher when compared to corresponding concentrations of colchicine-treated macrophages (*p <* 0.001 for all comparisons), and not significantly different from corresponding P6 peptide concentrations (*p >* 0.05 for all comparisons). The lack of a toxic effect from the colchicine-P6 peptide conjugate may be explained by the role of the P6 peptide in the uptake of colchicine by macrophages. Colchicine, due to its lipophilicity, is able to cross the cell membrane into the cytosol and is thus available to bind and disrupt microtubules in the cytosol. Colchicine can concentrate in cells due to its high binding affinity toward microtubules. This is evident by the ability of neutrophils from patients with gout to concentrate colchicine compared to levels found in patients’ sera [36,37]. Conjugated colchicine, on the other hand, is internalized by macrophages in a CD44 receptor-mediated mechanism. The internalization of CD44 with the colchicine-P6 peptide conjugate results in the formation of an endosome that later fuses with the lysosome [32]. In this uptake mechanism, colchicine does not concentrate in the cytosol and is thus unavailable to disrupt the microtubules and consequently interfere with key cellular processes (e.g., ATP synthesis resulting in cell death) [32,36,37].

### 2.9. Impact of P6 Peptide, Colchicine, and Colchicine-P6 Peptide Treatments on Monosodium Urate (MSU) Crystal Phagocytosis by Human THP-1 Macrophages

**Figure 5:**
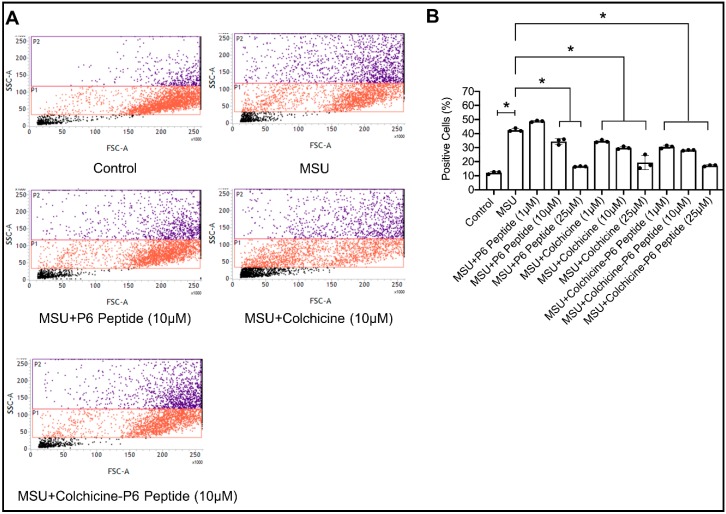
Impact of P6 peptide, colchicine, and colchicine-P6 peptide conjugate treatments on monosodium urate (MSU) crystal phagocytosis by human THP-1 macrophages. THP-1 macrophages were pre-treated with the P6 peptide, colchicine, or colchicine-P6 peptide (1, 10, or 25 μM) for 24 h followed by treatment with MSU crystals (100 μg/mL) for 4 h. Data are presented as scatter plots of three independent experiments with the mean and standard deviation highlighted. * p < 0.001. (**A**) Representative flow cytometry plots depicting increased macrophage cell population side-scatter in MSU-treated THP-1 macrophages, indicative of MSU phagocytosis. Treatments with P6 peptide, colchicine, or P6 peptide-colchicine (10 μM) appeared to reduce macrophage cell clustering in the P2 region indicative of reduced MSU phagocytosis. (**B**) P6 peptide (10 and 25 μM), colchicine (1, 10, and 25 μM) and P6 peptide-colchicine (1, 10, and 25 μM) treatments reduced MSU phagocytosis by THP-1 macrophages.

The impact of P6 peptide, colchicine, and colchicine-P6 peptide treatments on MSU phagocytosis by THP-1 macrophages is shown in Figure 5. Following a 4 h incubation, THP-1 macrophages internalized MSU crystals. P6 peptides (10 and 25 μM) significantly reduced MSU phagocytosis by THP-1 macrophages (Figure 5B, *p <* 0.001 for both comparisons). In addition, colchicine-P6 peptide treatments (1, 10, and 25 μM) reduced MSU phagocytosis to a comparable level as observed with the P6 peptide alone (*p <* 0.001 against MSU alone; *p >* 0.05 against MSU + P6 peptide). We also observed that colchicine treatments (1, 10, and 25 μM) reduced MSU phagocytosis as determined by flow cytometry. This effect may be related to the cytotoxic effect of colchicine against THP-1 macrophages. As a significant proportion of macrophages undergo apoptosis due to colchicine treatment, the number of macrophages available to internalize MSU crystals would be significantly reduced. As our assay measures the percentage of cells that internalized MSU crystals out of the whole cell population, it is imperative that the cell population remains viable. Therefore, it appears that the percentage of positive cells in colchicine-treated macrophages was artificially lowered due to the toxic effect of colchicine. The reduction in macrophage MSU phagocytosis with P6 peptide treatment is consistent with our finding that macrophage CD44 participates in MSU phagocytosis and that occupying the receptor with a high-molecular weight ligand (e.g., HA, PRG4 or in this case a small peptide) has a therapeutic effect in conditions of crystal-induced inflammation. Our findings support that the role of P6 in the colchicine-P6 peptide extends beyond modulation of colchicine off-target effect on microtubules, but also offers a pharmacologically-active role in the conjugate.

### 2.10. Anti-Inflammatory Efficacy of P6 Peptide, Colchicine, and Colchicine-P6 Peptide Treatments in MSU-Stimulated THP-1 Macrophages

**Figure 6:**
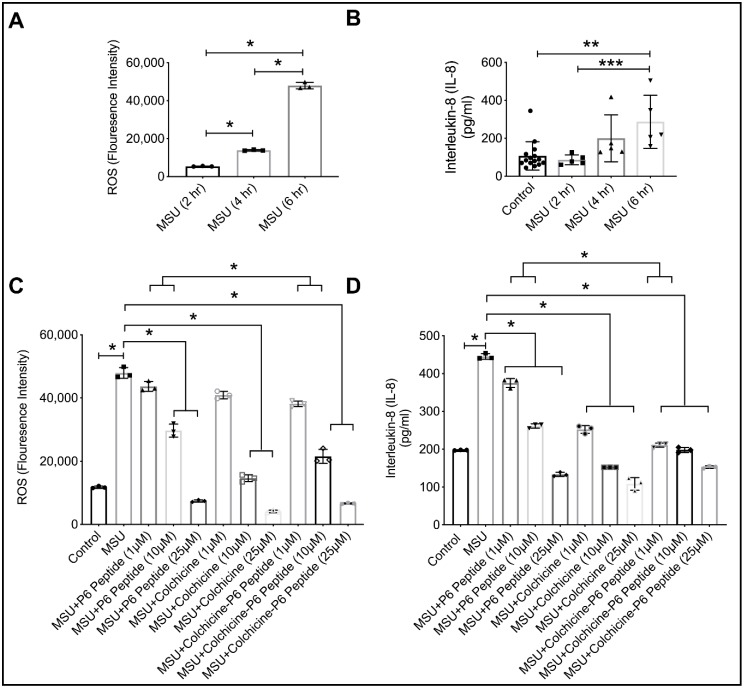
Time-dependent impact of monosodium urate (MSU) crystals on the generation of reactive oxygen species (ROS) and the production of inflammatory chemokine interleukin-8 (IL-8) by human THP-1 macrophages and anti-inflammatory efficacy of the P6 peptide, colchicine, and colchicine-P6 peptide in MSU stimulated THP-1 macrophages. Treatments with the P6 peptide, colchicine, and colchicine-P6 peptide (1, 10, and 25 μM) were performed for 24 h followed by the addition of MSU crystals (100 μg/mL) for 6 h followed by the determination of ROS generation by flow cytometry and IL-8 production by enzyme-linked immunosorbent assay (ELISA). Data are presented as scatter plots of three independent experiments with the mean and standard deviation highlighted. * p < 0.001; ** p < 0.01; *** p < 0.05. (**A**) MSU increased ROS generation at 4 and 6 h treatments compared to 2 h. (**B**) MSU increased IL-8 production by THP-1 macrophages at 6 h compared to 2 and 4 h. (**C**) Colchicine-P6 peptide was more efficacious than the P6 peptide alone at 1 and 10 μM in reducing ROS generation in THP-1 macrophages. (**D**) Colchicine-P6 peptide was more efficacious than the P6 peptide alone at 1 and 10 μM in reducing IL-8 production by THP-1 macrophages.

We initially studied the time-dependent ROS generation and IL-8 secretion following the incubation of THP-1 macrophages with MSU crystals (100 μg/mL) (Figure 6A,B). At 6 h, MSU induced a significant increase in intracellular ROS levels that corresponded to a significant increase in IL-8 secretion. P6 peptide treatments (10 and 25 μM) reduced ROS generation and IL-8 secretion (1, 10, and 25 μM) (Figure 6C,D; *p <* 0.001 for all comparisons against MSU alone). Colchicine-P6 peptide (1 μM and 10 μM) demonstrated superior efficacy in reducing ROS generation and IL-8 secretion compared to corresponding P6 peptide concentrations (Figure 6C,D; *p <* 0.001 when comparing MSU + colchicine-P6 peptide vs. MSU + P6 peptide). The mean estimated reduction in ROS due to MSU + colchicine-P6 peptide treatment compared to the MSU + P6 peptide treatment was 27.0% at 10 μM concentration. In addition, the mean estimated reduction in IL-8 secretion due to MSU + colchicine-P6 peptide treatment compared to MSU + P6 peptide treatment was 43.8% at a 1 μM concentration and 24.2% at a 10 μM concentration. The enhancement of the P6 peptide’s anti-inflammatory efficacy by colchicine conjugation was biologically significant at 1 and 10 μM concentrations. Interestingly, this enhancing effect disappeared at the 25 μM concentration due to the ceiling effect observed with the P6 peptide effect. Colchicine alone reduced ROS generation (10 and 25 μM) and IL-8 secretion (1, 10, and 25 μM). The superior efficacy of the colchicine-P6 peptide compared to P6 peptide alone is explained by the colchicine moiety, which in itself possesses the ability to reduce ROS generation and downstream IL-8 secretion.

### 2.11. Stability of P6 Peptide in Synovial Fluid (SF) Aspirates from Subjects with no History of Arthritis

We evaluated the time-dependent impact of normal SF (n = 3) incubation on P6 peptide stability and determined the half-life of the P6 peptide under this condition. At 1 h of incubation, the mean percentage of P6 peptide ± standard deviation (S.D.) recovered from SF was 47.6% ± 38.7% compared to 45.1% ± 14.7% at 2 h, 40.2% ± 28.8% at 3 h, 49.2% ± 7.8% at 4 h, 36.5% ± 6.2% at 6 h, 18.4% ± 1.3% at 12 h, and 6.4% ± 4.1% at 24 h (Figure S5, Supplementary Materials). The estimated half-life of the P6 peptide in normal SF was approximately 4 h. In this regard, the P6 peptide has a longer half-life compared to other peptides that bind surface antigens on immune cells, which showed a range of half-life values between 10–100 min in SF [48]. The high variability in the recovery of the P6 peptide from the SF aspirates might be related to the low number of unique aspirates we studied, coupled with reported high inter-donor variability [49]. The origin and composition of SF proteins are not entirely understood. A limited number of studies have attempted to systematically characterize the proteome of SF [50]. The SF proteome contains proteins secreted from cartilage, synovium, and proteins that originate from plasma. In fact, SF protein composition shares many similarities with the protein composition of plasma [50]. Therefore, the turnover of the P6 peptide is likely to be due to an intrinsic tryptic protease activity derived from plasma [51].

## 3. Materials and Methods

### 3.1. Materials

*N*-Deacetylcolchicine was purchased from DSK Biopharma, Inc. (Morrisville, NC, USA). All amino acids and resins were purchased from Advanced Automated Peptide Protein Technologies (APPTEC). All other chemicals and reagents were purchased from Sigma-Aldrich. The chemical structures of final products were confirmed by high-resolution MALDI-TOF (GT 0264) from Bruker Inc. and Bruker Impact II^TM^ UHR-QqTOF (Ultra-High Resolution Qq-Time-Of-Flight) mass spectrometry. Final compounds were purified by a reversed-phase HPLC from Shimadzu (LC-20AP) using a gradient system of acetonitrile and water and a reversed-phase preparative column (XBridge BEH130 Prep C18, Waters, Inc., Milford, MA, USA). Phorbol 12-myristate-13-acetate (PMA), soluble uric acid, high-binding microtiter plates (Corning), phosphate buffered saline (PBS), bovine serum albumin (BSA), fetal bovine serum (FBS), and Tween 20 were purchased from Sigma Aldrich (St. Louis, MO, USA). The CD44-IgFc fusion protein, HA, recombinant human IL-1β, IL-8 ELISA, and murine colony stimulating factor (M-CSF) was purchased from R&D Systems (Minneapolis, MN, USA). Anti-human IgG Fc-HRP, DyLight 488 goat anti-mouse IgG, anti-CD44 antibody, anti-α-Tubulin antibody, fluoroshield-mounting medium with DAPI, Cell Counting Kit-8 assay, and ROS assays were purchased from Abcam (Cambridge, MA, USA). Human THP-1 monocytes and RPMI-1640 media were purchased from American Type Culture Collection (ATCC) (Manassas, VA, USA). Pyrogen-free MSU crystals were obtained from Invivogen (San Diego, CA, USA). Penicillin and Streptomycin cocktail and Turbo TMB-ELISA substrate were obtained from ThermoFisher Scientific (Waltham, MA, USA). Normal SF aspirates were obtained from Articular Engineering (Northbrook, IL, USA) from three donors with no history of arthritis within 24 h of death, collected with partner site Institutional Review Board approval with informed consent from the nearest relative. *Cd44^+/+^* and *Cd44^−/−^* mice were obtained from Jackson Labs (Bar Harbor, ME, USA).

### 3.2. Methods

#### 3.2.1. Synthesis of P6 Peptide

Fmoc Rink amide resin (0.57 mmol/g, 0.52 g) was swelled in DMF for 15–30 min under dry nitrogen. The solvent (DMF) was filtered off after agitation. Piperidine in DMF (10 mL, 20% *v*/*v*) was used to remove the Fmoc protecting group. Then, the first Fmoc protected amino acid (Fmoc-Phe-OH) (4 equiv.) was coupled to the *N*-terminal of the resin using *N*,*N*,*N′*,*N′*-tetramethyl-O-(1H-benzotriazol-1-yl)uranium hexafluorophosphate, O-(benzotriazol-1-yl)-*N*,*N*,*N′*,*N′*-tetramethyluronium hexafluorophosphate (HBTU) (4 equiv.) and DIPEA (4 equiv.) in DMF under dry nitrogen for 1.5 h. Then, the reaction solution was filtered out after completion of the coupling. The resin was washed off by DMF (10 mL, 3 × 2 min.). Fmoc deprotection was done by adding piperidine in DMF (20% *v*/*v*, 10 mL, 3 × 10 min). The reaction solution was filtered out and washed again with DMF. The subsequent Fmoc-protected amino acids were coupled to the peptide template until the last amino acid (Fmoc-Trp(Boc)-OH) was coupled. The final Fmoc deprotection was carried out to make a free *N*-terminal for the conjugation of colchicine. Cleavage cocktail containing TFA, thioanisole, EDT and anisole (90:5:3:2 *v*/*v*/*v*/*v*, respectively, 6 mL) was used to cleave the resin and all side chain-protecting groups form the peptide to check the final sequence. The mixture was stirred at room temperature for 4 h. Then, the crude conjugate was precipitated using cold diethyl ether and centrifuged at 10 G for 10 min. The precipitant was dissolved in water containing 0.1% TFA and acetonitrile containing 0.1% TFA in an equal amount (1:1 *v*/*v*). After completion of the synthesis of the 20 amino acid sequence of the P6 peptide, the linear peptide was confirmed by MALDI analysis (m/z): C_115_H_165_N_26_O_30_ calculated for M + H. 2390.2185, found 2389.9299 [M + H]^+^.

#### 3.2.2. Synthesis of Colchicine-Glutaryl Amide Conjugate

Coupling of *N*-deacetylcolchicine (35 mg, 0.098 mmol) with glutaric anhydride (11.97 mg, 0.105 mmol) was carried out by solution-phase chemistry in the presence of anhydrous tetrahydrofuran (THF, 10 mL), 4-dimethylaminopyridine (DMAP, 8.5 mg, 0.07 mmol), and triethylamine (TEA, 31.5 μL, 0.23 mmol) as a base. The reaction mixture was stirred under nitrogen for 4 h. The solvent (THF) was removed by a rotatory evaporator under reduced pressure and was then dissolved using DCM. Hexane was used to crystallize the product (30 mg, 64%). The compound was used in conjugation with resin-attached P6 peptide to afford the desired conjugate with the P6 peptide. Q-TOF analysis (m/z): C_25_H_30_NO_8_ Calculated, 472.1971 for M + H; Found 472.2045 [M + H]^+^, 494.1869 [M + Na]^+^, 510.1610 [M + K]^+^).

#### 3.2.3. Synthesis of Colchicine-P6 Peptide Conjugate

The peptide-attached resin (230 mg) was used in the coupling with the colchicine-glutaryl amide conjugate (30 mg, 0.075 mmol) synthesized by solution-phase chemistry. Anhydrous DMF (10 mL), PyBOP (benzotriazol-1-yl-oxytripyrrolidinophosphonium hexafluorophosphate (58.5 mg, 0.11 mmol)), 1-hydroxybenzotriazole hydrate (HOBt, 30.3 mg, 0.22 mmol), and *N*, *N*-diisopropylethylamine (DIPEA, 80 μL, 0.075 mmol) were added to the reaction mixture of the peptide-attached resin and colchicine-glutaryl amide conjugate in 1 mL anhydrous DMF. This reaction mixture was shaken on a rotator overnight. After the formation of the conjugate was confirmed by MALDI analysis, the crude conjugate was washed for 2 h using DMF and DCM. The cleavage cocktail containing TFA, thioanisole, EDT and anisole (90:5:3:2 *v*/*v*/*v*/*v*, respectively, 6 mL) was used to cleave the resin and all side chain-protecting groups form the conjugate. The mixture was stirred at room temperature for 4 h. Then, the crude conjugate was precipitated using cold diethyl ether and centrifuged at 10 G for 10 min. The precipitant was dissolved in water containing 0.1% TFA and acetonitrile containing 0.1% TFA in an equal amount (1:1 *v*/*v*). After purification via reversed-phase HPLC and lyophilization (3 mg), the structure of the conjugate was confirmed by high-resolution MALDI-TOF analysis (m/z): C_140_H_192_N_27_O_37_ Calculated, 2843.3972 for M + H, found 2843.8766 [M + H]^+^.

#### 3.2.4. Synthesis of Fluorescein-Labeled P6 Peptide

Protected P6 peptide-attached resin (170 mg) was used in the coupling with 5(6)-carboxyfluorescein diisobutyrate on a solid support. Anhydrous DMF (10 mL), PyBOP (39 mg, 0.075 mmol), HOBt (20 mg, 0.15 mmol), and DIPEA (40 μL, 0.23 mmol) in 1 mL anhydrous DMF was added to the reaction mixture of crude protected peptide-attached resin and 5(6)-carboxyfluorescein diisobutyrate (38.5 mg, 0.072 mmol). This reaction was shaken on a rotator overnight. After the formation of the compound was confirmed by MALDI analysis, the resin was washed for 2 h using DMF and DCM. The cleavage cocktail containing TFA, thioanisole, EDT, and anisole (90:5:3:2 *v*/*v*/*v*/*v*, respectively, 5 mL) was used to cleave the resin and all side-chain protecting groups from the compound. The mixture was stirred at room temperature for 4 h. Then, the crude was precipitated using cold diethyl ether and centrifuged at 10 G for 10 min. The precipitant was dissolved in water containing 0.1% TFA and acetonitrile containing 0.1 TFA in an equal amount (1:1 *v*/*v*) and was purified by reversed-phase HPLC. MALDI-TOF analysis (m/z): C_137_H_179_N_26_O_35_ Calculated. 2750.0860 for M + H, found 2749.6489 [M + H]^+^.

#### 3.2.5. Differentiation of THP-1 Macrophages and CD44 Expression under Conditions of Inflammation, Isolation of BMDMs from Cd44^+/+^ and Cd44^−/−^ Animals and Impact of HA Treatment on MSU Phagocytosis by THP-1 Macrophages

THP-1 monocytes were cultured in RPMI 1640 medium supplemented with 10% heat-inactivated FBS, 10 mM HEPES, 2 mM glutamine, 100 U/I penicillin, and 100 μg/mL streptomycin and maintained at 37 °C and 5% CO_2_. THP-1 monocytes were differentiated into THP-1 macrophages using 50 ng/mL phorbol-12-myristate 13-acetate (PMA) for 24 h [34]. Subsequently, adherent cells were washed twice with serum-free medium and rested for another 24 h in PMA-free RPMI + 10% FBS medium. CD44 expression on THP-1 macrophages was conducted using flow cytometry following incubation with the CD44-specific antibody (1:1000 dilution) for 1 h followed by cell pelleting and washing the cell pellet with PBS. THP-1 macrophages were then incubated with DyLight 488 goat anti-mouse IgG at 1:1000 dilution for 30 min. Following cell pelleting and washing, cells were fixed in 4% paraformaldehyde and cell-associated fluorescence was determined by flow cytometry using BD FACSVerse (BD Biosciences). THP-1 macrophages (500,000 cells per well) were treated with IL-1β (10 ng/mL) or uric acid (10 mg/dL) in serum-free RPMI 1640 medium for 24 h. THP-1 macrophages were washed with PBS, collected, and CD44 expression was determined using flow cytometry as described above.

*Cd44^+/+^* and *Cd44^−/−^* male mice (12–14 weeks old, n = 2 in each group) were euthanized, and isolation and culture of bone marrow derived macrophages (BMDMs) were performed as previously described using 10 ng/mL M-CSF [52]. BMDMs were seeded onto cover slips in 6-well plates (500,000 cells per well) and incubated with MSU crystals (100 μg/mL) for 4 h, followed by cell staining using fluoroshield-mounting medium with DAPI and visualized under a microscope. All animal experiments were approved by the Institutional Animal Care and Use Committee (IACUC) at Chapman University.

THP-1 macrophages (500,000 cells per well) were treated with MSU crystals (100 μg/mL) ± HA (100 μg/mL) for 4 h. Subsequently, macrophages were collected, pelleted, and washed with PBS. MSU phagocytosis was determined by analyzing the change in cell side-scatter using a flow cytometer. Two regions of interest were identified: the P1 region representing the THP-1 macrophage population in the absence of MSU crystal exposure and the P2 region with increased cell side scatter indicative of MSU phagocytosis. Data are presented as the percentage of cells in the P2 region across different experimental groups.

#### 3.2.6. Comparative Binding of P6 Peptide and HA Using an Enzyme-Linked Immunosorbent Assay (ELISA) Format

Wells of high-binding microtiter plates were coated with either the P6 peptide or high molecular weight HA (molecular weight >1 million Da) in serial concentrations between 1 ng/mL and 1 μg/mL overnight in phosphate buffered saline (PBS) (100 µL per well) at 4 °C. To remove unbound molecules, wells were washed with PBS + 0.05% Tween 20 and subsequently blocked using 2% bovine serum albumin (BSA) (300 µL per well) for 2 h at room temperature. CD44-IgG Fc fusion protein (R&D Systems) (0.1 µg/mL) in PBS + 0.05% Tween 20 (100 µL per well) was added to both coated and uncoated wells and incubated for 1 h at room temperature. Following washing with PBS + 0.1% Tween 20, anti-human IgG Fc-HRP (1:1000) was added and incubated at room temperature for 1 h, followed by washing three times with PBS + 0.05% Tween 20. Turbo TMB-ELISA substrate solution was added (100 µL per well), and absorbance intensity was determined at 450 nm. Absorbance values in the P6 peptide and HA-coated wells were subtracted from the absorbance values of uncoated wells to adjust for any non-specific binding by CD44-IgFc.

#### 3.2.7. CD44-Dependent Association and Internalization of Fluorescein-P6 Peptide in THP-1 Macrophages

THP-1 macrophages (500,000 cells per well) were treated with different concentrations of the fluorescein-labeled P6 peptide (1, 10, or 25 μM) for 30 or 60 min, after which cells were washed with PBS, trypsinized, and fixed using 4% paraformaldehyde. Cell-associated fluorescence was determined by flow cytometry using BD FACSVerse. In a separate set of experiments, THP-1 macrophages were pretreated with anti-CD44 antibody (2.5 μg/ml) for 1 h prior to the addition of fluorescence-labeled P6 peptide (1, 10 or 25 μM) for 1 h. Cell-associated fluorescence was determined as described above.

THP-1 macrophages (250,000 cells per well) were seeded in 4-well chamber slides followed by treatment with different concentrations of the fluorescein-labeled P6 peptide (1, 10, or 25 μM) for 6 h. Subsequently, cells were fixed using 4% paraformaldehyde for 10 min followed by washing with PBS and permeabilization with 0.1% Triton X-100 for 5 min. Following washing with PBS, cells were blocked for 30 min in 2% BSA in PBS. Cells were then incubated with anti-α tubulin monoclonal antibody (1:1000) for 2 h. Nuclear staining was performed using 4,6-diamidino-2-phenylindole (DAPI). Confocal imaging and Z-stacking were performed using a Nikon Ti-E microscope. Intracellular mean fluorescence was determined using three different fields in each group.

#### 3.2.8. Cytotoxicity of P6 Peptide, Colchicine, and Colchicine-P6 Peptide against THP-1 Macrophages

THP-1 macrophages were plated in a 96-well plate at a density of 1 × 10^4^ per well and treated with different concentrations of P6 peptide, colchicine, colchicine-P6 peptide conjugate (1, 10, or 25 μM) for 24 h. Subsequently, 10 μL of CCK-8 solution was added to each well and absorbance was measured 90 min later at 450 nm. Cytotoxicity was expressed as the percentage of viable cells following treatments.

#### 3.2.9. Impact of P6 Peptide, Colchicine, and Colchicine-P6 Peptide Treatments on MSU Phagocytosis by THP-1 Macrophages and Downstream ROS Generation and IL-8 Secretion

THP-1 macrophages (500,000 cells per well) were incubated with the P6 peptide, colchicine, or colchicine-P6 peptide at 1, 10, or 25 μM concentrations for 24 h. Subsequently, cells were treated with MSU crystals (100 μg/mL) for 4 h. MSU phagocytosis by THP-1 macrophages was determined using flow cytometry as described above.

THP-1 macrophages (500,000 cells per well) were treated with MSU crystals (100 μg/mL) for 2, 4, and 6 h. ROS staining solution (1:1000 ratio) was added to the wells and incubated for 1 h. Subsequently, cells were collected, washed, and cell-associated fluorescence was determined using flow cytometry. Media were collected at 2, 4, and 6 h, and IL-8 media concentrations were determined by ELISA. In another set of experiments, THP-1 macrophages were incubated with the P6 peptide, colchicine, or colchicine-P6 peptide at 1, 10, or 25 μM concentrations for 24 h. Subsequently, cells were treated with MSU crystals (100 μg/mL) for 6 h. ROS generation and IL-8 concentrations were determined as described above.

#### 3.2.10. Stability of P6 Peptide in Normal Synovial Fluid (SF) Aspirates

P6 peptide stability in the presence of normal SF from three donors with no history of arthritis was examined using an analytical reversed-phase high-performance liquid chromatography (RP-HPLC). Using seven microcentrifuge tubes, a total of 50 μL of normal SF was diluted with 150 μL of PBS in each tube. Following equilibrating diluted SF to 37 ± 1 °C for 15 min, 50 μL of peptide stock solution (1 mM solution in 100% sterile water) was added to each tube. Next, at different time intervals (0, 1, 2, 3, 4, 6, and 24 h), an aliquot of the reaction solution (100 μL) was sampled. Cold methanol (200 μL) was added to each aliquot in order to precipitate proteins that were present in the SF. This was followed by cooling the solution to 4 °C for 15 min followed by centrifugation at 500 g for 15 min to precipitate SF proteins. To determine the proteolytic stability of the P6 peptide, the supernatant was injected into an analytical RP-HPLC C18 column. In order to measure the concentration of the P6 peptide isolated from SF, the area under the curve (AUC) of the P6 peptide at each time point was estimated using an average of four runs, and normalized to AUC values at time 0 and expressed as the percentage of AUC at time 0.

#### 3.2.11. Statistical Analyses

Data were derived from at least three independent experiments with a minimum of duplicate runs per group per experiment. Statistical significance comparing two groups or multiple groups was assessed by the Student’s *t*-test or analysis of variance (ANOVA) followed by post-hoc pairwise comparisons using Tukey’s post-hoc test. A *p* value of <0.05 was considered statistically significant.

## 4. Conclusions

In this study, we provided evidence supporting that CD44 expression is enhanced under conditions characteristic of gout and that the CD44 receptor is directly involved in initiating inflammation by MSU crystals in macrophages via facilitating the phagocytosis of MSU crystals. A 20 amino acid peptide ligand of CD44, the P6 peptide, showed a similar binding profile to CD44 as that of HA and the association and internalization of the P6 peptide by human THP-1 macrophages was CD44-dependent. We successfully synthesized a colchicine-P6 peptide conjugate with a hydrolyzable glutaryl amide linker. The conjugation of colchicine to the P6 peptide abolished the cytotoxic effect of colchicine against THP-1 macrophages at a concentration range between 1 and 25 μM. The colchicine-P6 peptide reduced MSU phagocytosis by THP-1 macrophages due to the ability of the P6 peptide moiety to bind CD44, thus attenuating CD44′s role in mediating MSU phagocytosis. On the other hand, the colchicine-P6 peptide conjugate showed superior efficacy in reducing ROS generation and IL-8 secretion, potentially due to the contribution of the colchicine moiety where colchicine was efficacious in reducing ROS generation and downstream inflammatory chemokine production. The stability of the P6 peptide in normal SF was better than previously reported peptides that bind immune cells in SF. Taken together, conjugation of colchicine to the P6 peptide minimized the off-target cytotoxic effect of colchicine and preserved the anti-inflammatory effect of colchicine with the P6 peptide moiety playing an effector role in reducing MSU phagocytosis and downstream inflammation in macrophages. The colchicine-P6 peptide is a potential novel therapeutic in gout and future work will evaluate the kinetics and efficacy of the intra-articular colchicine-P6 peptide in an animal model of acute gouty arthritis. To our knowledge, this is the first report demonstrating the utility of a colchicine-peptide conjugate as an anti-inflammatory therapeutic.

## Supplementary Materials

The MALDI spectra of selected compounds and P6 peptide stability in normal SF aspirates are provided.
