# Supplementary material for: Design and Biological Evaluation of Colchicine-CD44-Targeted Peptide Conjugate in an In Vitro Model of Crystal Induced Inflammation
Source: Molecules. 2019 Dec 21;25(1):46. doi: 10.3390/molecules25010046 (PMC6982808; doi:10.3390/molecules25010046)
Supplement: Supplementary file 1 [file molecules-25-00046-s001.pdf]

Supplementary Materials

Design and Biological Evaluation of Colchicine-CD44-Targeted Peptide Conjugate in an *In-Vitro* Model of Crystal Induced Inflammation

Khalid A. Zoghebi[1,2,#], Emira Bousoik[1,#], Keykavous Parang[1,*] and Khaled A. Elsaid [1,*].

[1] Center for Targeted Drug Delivery, Department of Biomedical and Pharmaceutical Sciences, Chapman University School of Pharmacy, Harry and Diane Rinker Health Science Campus, Irvine, CA 92618, United States; bouso100@mail.chapman.edu (E.B); zoghe101@mail.chapman.edu (K.A.Z); parang@chapman.edu (K.P); elsaid@chapman.edu (K.A.E).

[2] Department of Pharmaceutical Chemistry, College of Pharmacy, Jazan University, Jazan 82826, Saudi Arabia; kzoghebi@jazanu.edu.sa

*Correspondence: parang@chapman.edu (K.P.); Tel.:+1-714-516-5489; elsaid@chapman.edu (K.A.E.) +1 (714) 516-5435

[#]Both authors contributed equally.

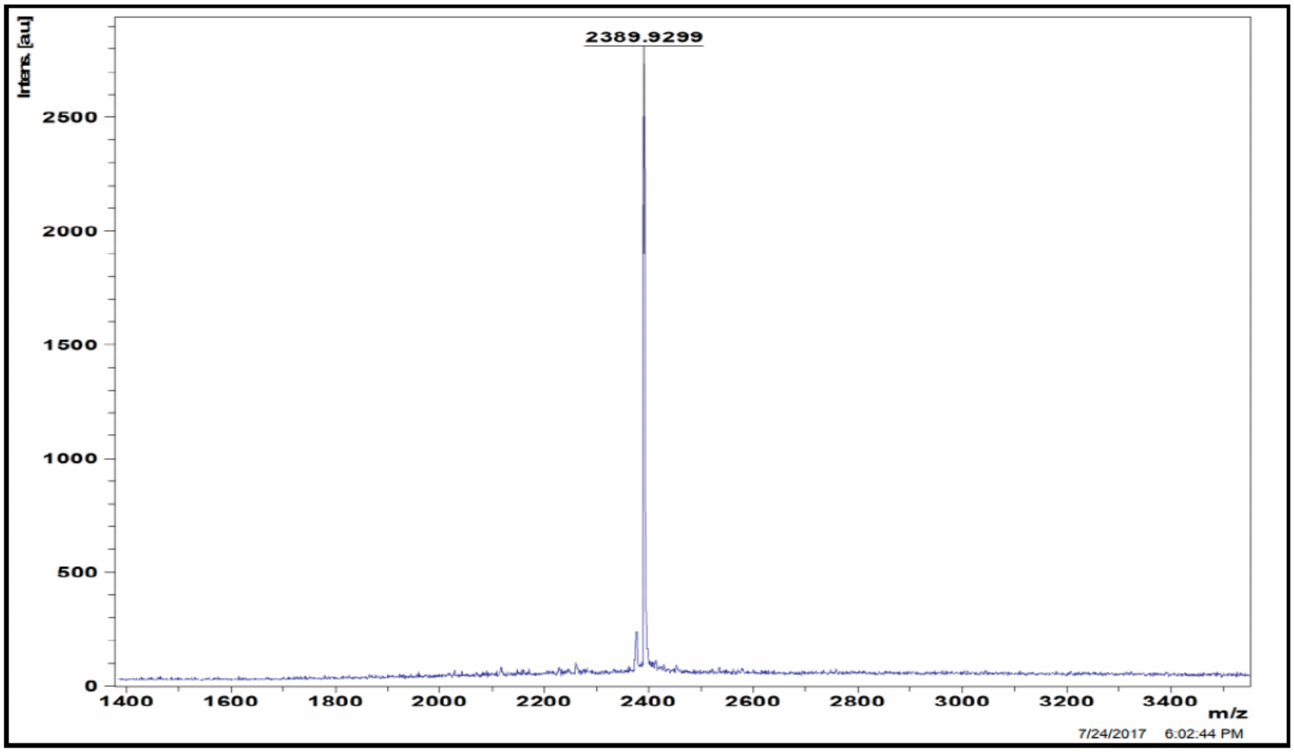

Figure S1. MALDI/TOF (m/z) mass spectroscopy for pure P6 peptide. The $[M + H]^+$ value is shown (MALDI analysis (m/z): $C_{115}H_{165}N_{26}O_{30}$ calculated for M + H. 2390.2185, found 2389.9299 $[M + H]^+$).

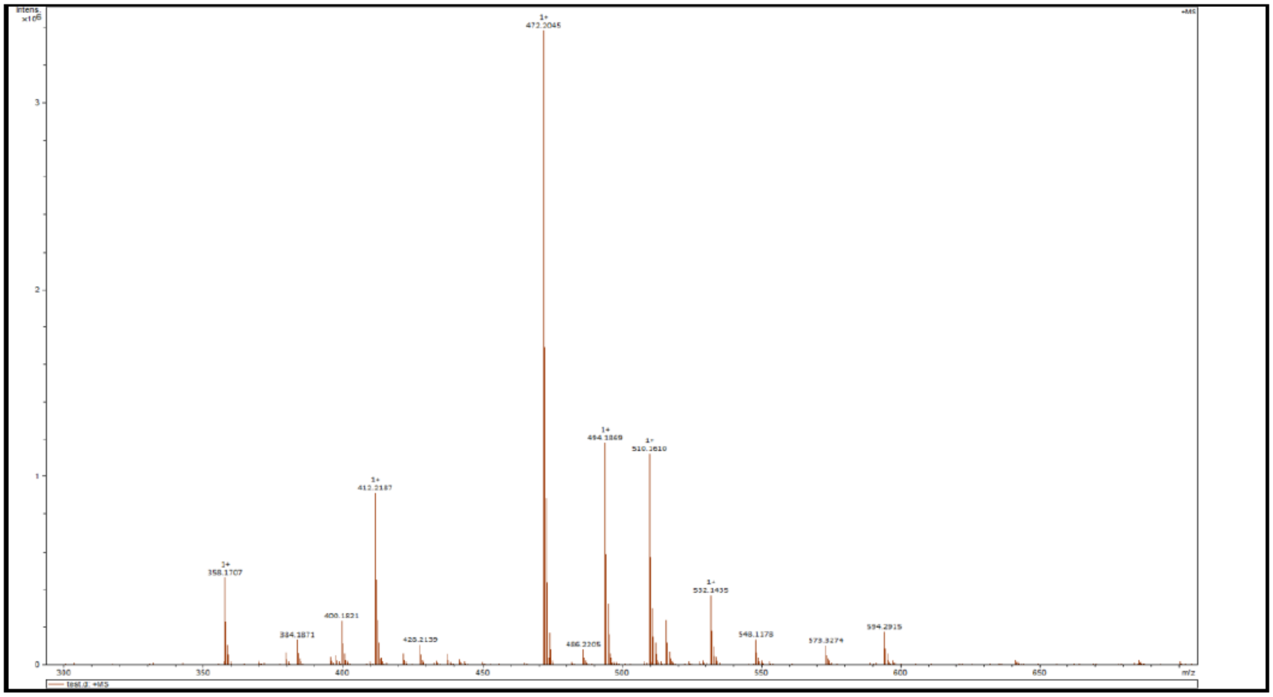

Figure S2. Q-TOF (m/z) mass spectroscopy for crude colchicine-glutaryl amide (Q-TOF analysis (m/z): $C_{25}H_{30}NO_8$ Calculated, 472.1971 for M + H; Found 472.2045 $[M + H]^+$, 494.1869 $[M + Na]^+$, 510.1610 $[M + K]^+$).

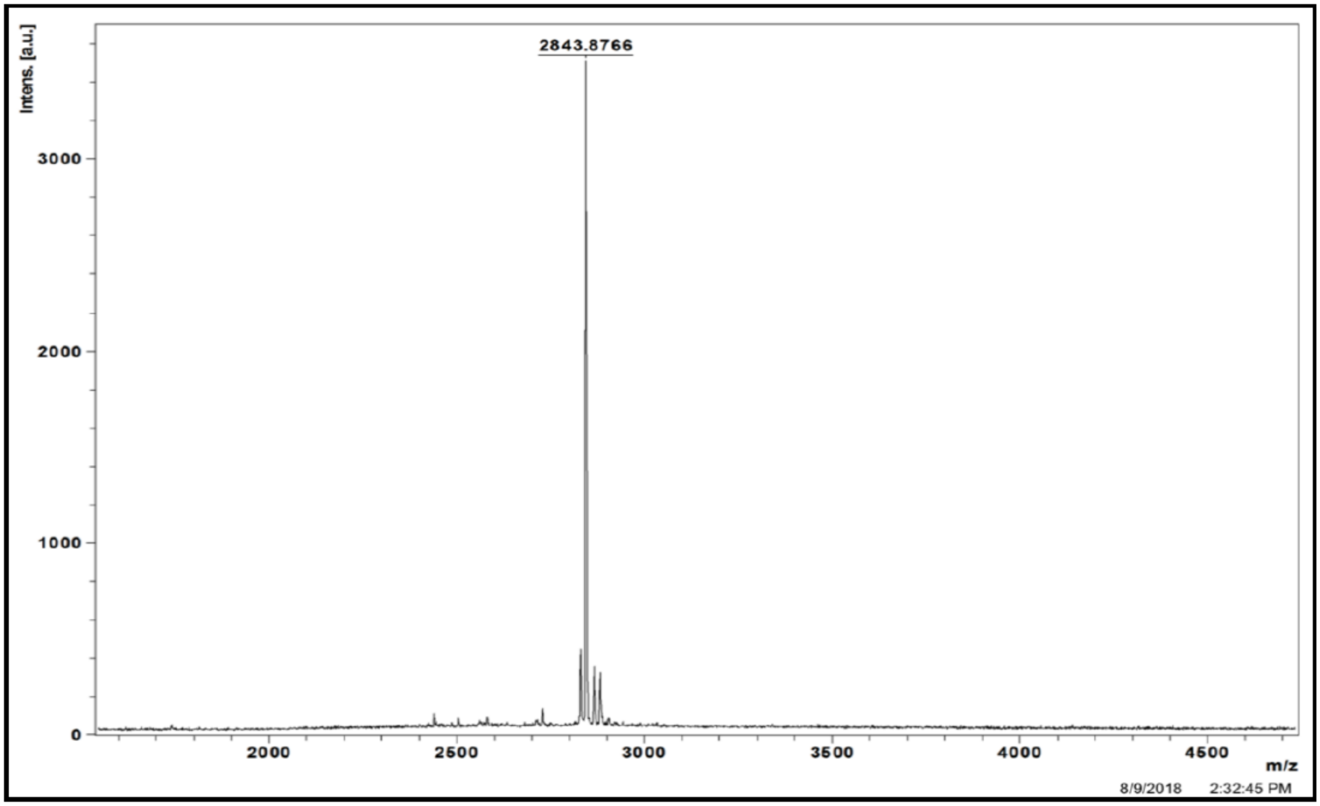

Figure S3. MALDI-TOF (m/z) mass spectroscopy for pure colchicine-P6 peptide conjugate (MALDI-TOF analysis (m/z): $C_{140}H_{192}N_{27}O_{37}$ Calculated, 2843.3972 for M + H, found 2843.8766 $[M + H]^{+}$).

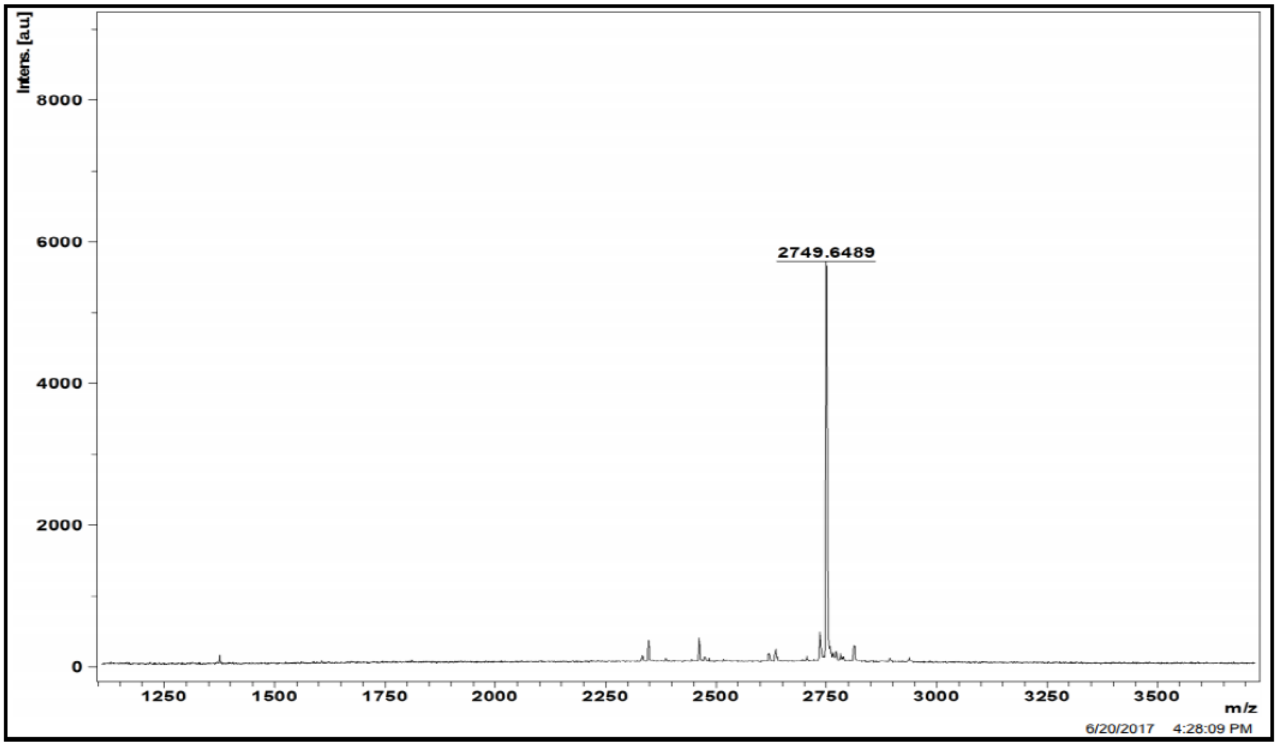

Figure S4. MALDI-TOF (m/z) mass spectroscopy for pure fluorescein-labeled P6 peptide (MALDI-TOF analysis (m/z): $C_{137}H_{179}N_{26}O_{35}$ Calculated. 2750.0860 for M + H, found 2749.6489 $[M + H]^+$).

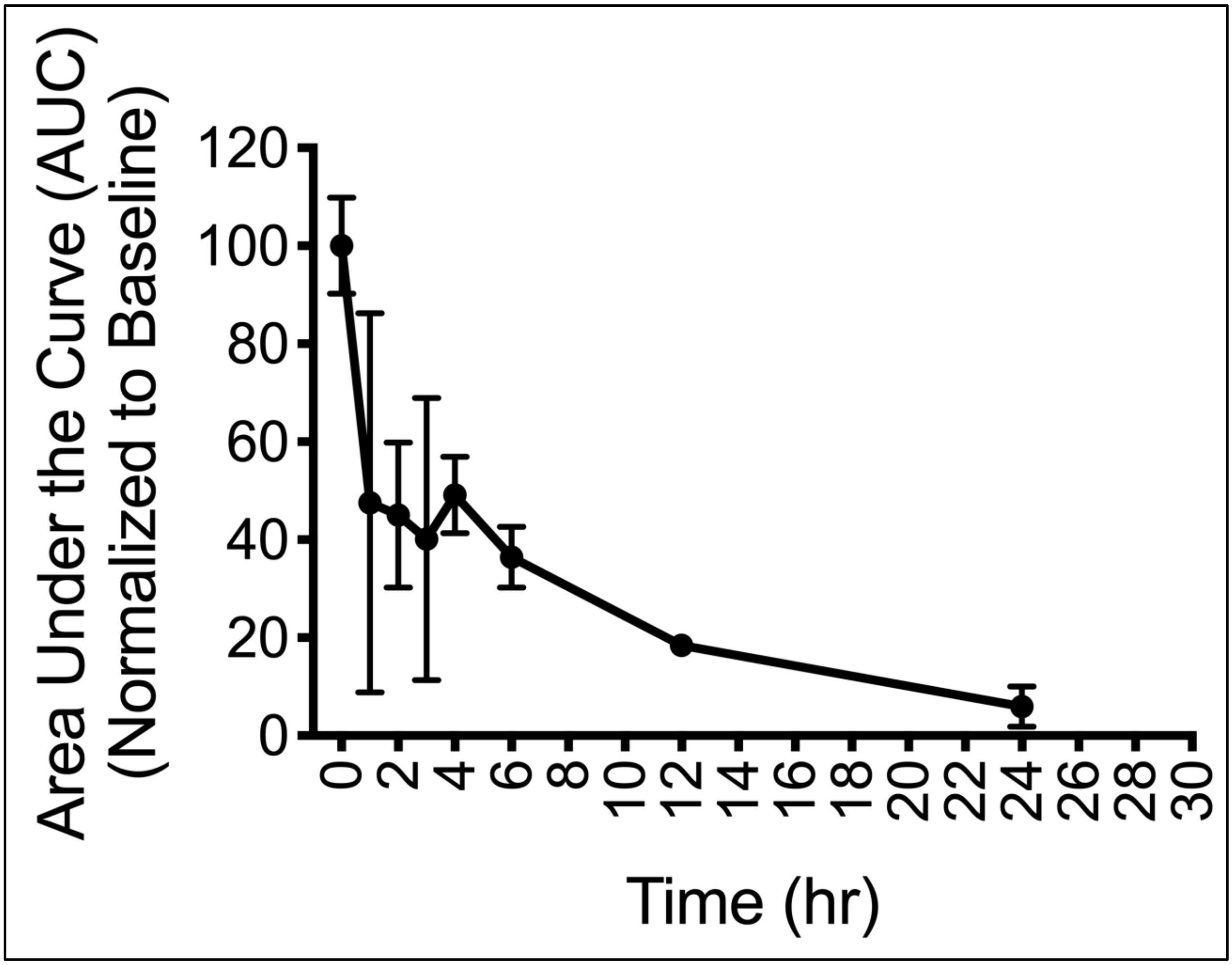

Figure S5. Time-dependent degradation of P6 peptide following incubation with synovial fluid (SF) aspirates from subjects with no history of degenerative joint diseases (n=3). Determination of P6 peptide concentrations in SF samples was performed using an HPLC assay and area under the curve (AUC) of the P6 peptide peak was determined and normalized to the mean AUC of P6 peptide at baseline (time = 0 hr). The estimated half-life of P6 peptide in normal SF was 4 approximately hours.
